# Lower-Limb Range of Motion Predicts Sagittal Spinal Misalignments in Children: A Case-Control Study

**DOI:** 10.3390/ijerph19095193

**Published:** 2022-04-25

**Authors:** Antonio Cejudo

**Affiliations:** 1 Department of Physical Activity and Sport, Faculty of Sport Sciences, CEIR Campus Mare Nostrum (CMN), University of Murcia, 30720 Murcia, Spain; antonio.cejudo@um.es; Tel.: +34-868-888-430; 2 Locomotor System and Sport Research Group (E0B5-07), University of Murcia, 30720 Murcia, Spain

**Keywords:** limited range of motion, iliopsoas tightness, hamstring tightness, ROM-SPORT battery, lower back pain, prevention of back pain, school-age children

## Abstract

The main objective of this study was to determine lower-limb range of motion (ROM) as a risk factor associated with sagittal spinal misalignments in children. Two hundred and one children (10.9 ± 0.7 years old) from five different primary schools were recruited for this retrospective case—control study. Anthropometric variables, sagittal spinal alignment in habitual everyday postures, and lower-limb ROM, such as ankle dorsiflexion with the knee flexed (ADF-KF), hip extension with the knee relaxed (HE), and hip flexion with the knee extended (HF-KE) were measured. Multivariate and univariate analyses revealed differences between the means of HE and HF-KE ROM, and the thoracic and lumbar curves (*p* ≤ 0.034; η^2^ ≥ 0.052). The HE (*p* ≤ 0.028; OR ≥ 1.066) predicted sagittal thoracic misalignment in the slump sitting (SSP) and relaxed standing (RSP) postures in males and the sagittal lumbar misalignment in the RSP in female children; while HF-KE (*p* ≤ 0.006; OR ≥ 1.089) predicted sagittal thoracic misalignment in the maximum trunk forward flexion posture (MTFP) and sagittal lumbar misalignment in SSP and MTFP in males. In this study, the reference values for restricted HE and HF-KE were significantly associated with sagittal spinal misalignment in male children but not for the ankle ROM. Physical education teachers should include stretching exercises in the ISQUIOS programme to increase the extensibility of the iliopsoas (HE) and hamstrings (HF-KE) and prevent sagittal spinal misalignments in habitual everyday postures.

## 1. Introduction

Epidemiological studies have reported high prevalence rates (up to 71%) of low back pain (LBP) in children, which are higher in older and female children [1,2]. LBP is associated with disability in a high percentage of children and subsequently leads to school absenteeism, reduced physical activity, and limited daily activities [3].

LBP is a multifactorial dysfunction. The hip is one of the potential biomechanical risk factors for LBP. The hip has a biomechanical connection with the pelvis and the spine [4,5,6]. The movements of the hip, pelvis, and lumbar spine are closely interconnected and strictly coordinated. The interplay between the hip and pelvis is commonly referred to as the pelvifemoral rhythm [4] and that between the lumbar spine and pelvis as the lumbopelvic rhythm [5]. The abnormal and asynchronous lumbar–pelvic–hip patterns, the lumbopelvic rhythm, or the pelvifemoral rhythm lead to excessive loading of the lumbar and hip joints when performing trunk movements resulting in injury and pain [7,8]. For example, pelvic retroversion during active hip flexion is increased in individuals with femoroacetabular impingement [4,9] or pelvic retroversion during trunk flexion, resulting in greater spinal loading and consequently causing LBP [8]. A restricted hip range of motion (ROM) due to muscle tightness and other joint tissues contributes to the faulty lumbopelvic or pelvifemoral rhythm and consequently to spinal misalignments and LBP [10,11]. Iliopsoas tightness (restricted hip extension) increases pelvic anteversion and causes lumbar hyperlordosis in standing [10]. Lumbar hyperkyphosis due to restricted pelvic anteversion and thoracic hyperkyphosis are caused by hamstring tightness (restricted hip flexion) during standing movements [6] and maximal trunk flexion [12,13].

Knowledge of the relationship between lower-limb ROM and sagittal spinal alignment in habitual everyday postures in children has not yet been investigated. The main aim of the current study was to investigate the hip and ankle ROM as risk factors associated with sagittal spinal misalignment in habitual everyday postures in children aged 10–12 years. The hypothesis is that a restrictive hip ROM predicts sagittal spinal misalignment, but the ankle joint ROM does not.

## 2. Materials and Methods

### 2.1. Study Design

An analytical, retrospective case-control observational study was conducted to investigate hip and ankle ROM as risk factors associated with sagittal spinal misalignment in habitual everyday postures in schoolchildren. This study followed the guidelines and checklist for case-control studies of Strengthening the Reporting of Observational Studies in Epidemiology (STROBE) [14]. The experimental procedures used in this study complied with the Declaration of Helsinki and were approved by the Ethics and Science Committee of the University of Murcia (ID: 1702/2017). Before participating in the study, teachers, parents, and children were informed both verbally and in writing about the experimental procedures and possible risks, and their written informed consent was obtained. The study was tightly controlled by keeping the expert and the children blinded to the objectives of the study.

To be included in the study, the children had to regularly attend physical education classes and the ISQUIOS program. ISQUIOS is a physical conditioning (muscle flexibility and core strength) and postural hygiene programme used in physical education classes. The aim of this intervention is to improve sagittal spinal alignment in the habitual everyday postures (sitting, standing, and trunk flexion) of schoolchildren and to provide guidance on the correct use and weight control of school bags.

Children were excluded if they (1) had their first menstrual period, (2) had back, pelvic and lower-limb pain, illness, or injury at the time of testing, (3) had orthopaedic problems that prevented proper performance of a test, and (4) had previously been diagnosed with scoliosis or treated for a spinal condition. Children from the ISQUIOS programme who had not completed a test were also excluded.

The specific criteria for cases were those children with sagittal thoracic and lumbar misalignment (increased sagittal spinal curves).

The tests were performed before the ISQUIOS programme. The children were instructed to avoid strenuous activity 48 h before the test. A familiarisation session was conducted with the participants one week before the tests. The testing procedure was conducted in a sports pavilion under standard conditions of 25 °C. The measurements were taken simultaneously by an examiner with more than 15 years of experience in musculoskeletal assessment. An assistant examiner helped the main examiner during the ROM tests to avoid compensatory movements.

Sagittal spinal alignment or dependent variable (study onset/outcome) was measured during the first semester of the 2018–2019 academic year. Anthropometric variables and lower-limb ROM (exposure/past) were measured retrospectively during the first semester of the 2017–2018 year (independent variables). The confounding variables were anthropometric data and gender. The tests were performed in random order to avoid bias in the results due to a particular order. Each test was performed three times and the average of the nearest measurements was calculated.

### 2.2. Sample

A sample of 201 children aged 10–12 years (103 males and 98 females; mean ± SD age, 10.9 ± 0.7 years; stature, 148.4 ± 7.3 cm; weight mass, 45.7 ± 11.6 kg) from five different public primary schools was used (Table 1). These schools had been selected to participate in the ISQUIOS program, a postural education program. The study was conducted during the first semester of the 2017–2018 and 2018–2019 school years.

### 2.3. Procedures

#### 2.3.1. Anthropometric Measurements

Anthropometric measurements were obtained using standardised techniques according to the protocol of ISAK manual [15]. Stature was measured with a mobile stadiometer (Seca 213; Seca Ltd., Hamburg, Germany) with an accuracy of 0.1 cm. Weight mass was measured with an electronic scale OMRON BF 500 (Omron Corp., Kyoto, Japan) with an accuracy of 0.1 kg.

#### 2.3.2. Measurements of Sagittal Spinal Alignment

**Figure 1:**
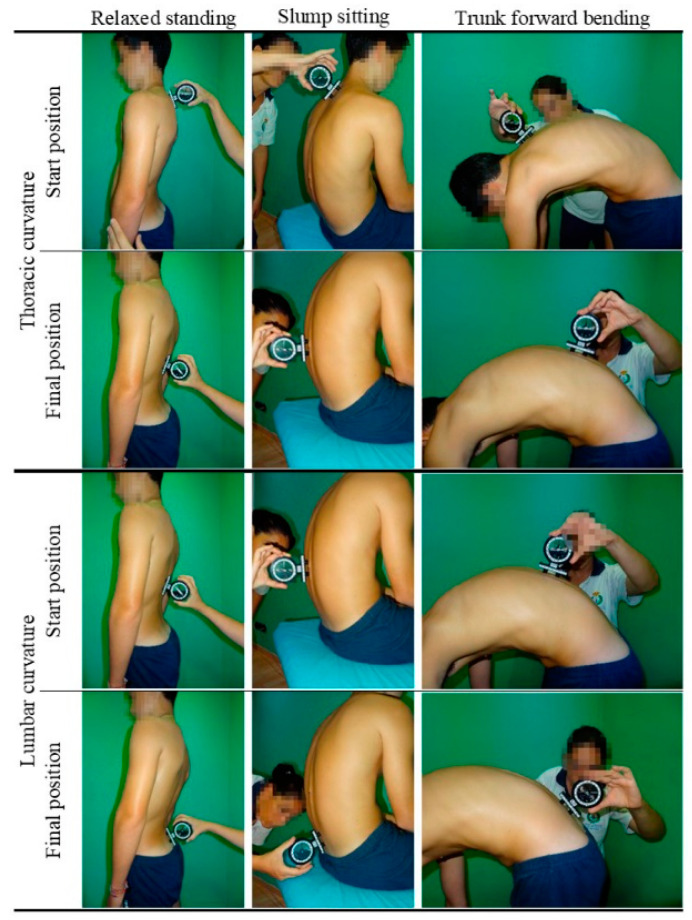
Thoracic and lumbar sagittal curves assessment tests.

Following the previously described method [16], the sagittal spinal alignment (thoracic and lumbar curves) or morphotype was assessed in the main positions that children may adopt in everyday life, such as slump sitting posture (SSP), the relaxed standing posture (RSP) and the maximum trunk forward flexion posture (MTFP) (Figure 1). An inclinometer (ISOMED, Inc., Portland, OR, USA) was used to quantify the sagittal spinal curves, which has high reproducibility and validity and shows a good correlation with radiography [17].

#### 2.3.3. Measurements of ROM

Ankle dorsiflexion with the knee flexed (ADF-KF) for the soleus and other joint tissues, hip extension with the knee relaxed (HE) for the iliopsoas, and hip flexion with the knee extended for the hamstring (HF-KE) of both the right and left limb were assessed using the ROM-SPORT method [18]. The ROM-SPORT was used in this study due to its reliability and validity based on sports experience and biomechanical knowledge [18,19,20].

An inclinometer (ISOMED, Inc., Portland, OR, USA) was used to quantify the ROM. The inclinometer was calibrated to 0° with either the vertical (ADF-KF) or the horizontal (HE and HF-KE) before the start of the study. The angle between the longitudinal axis of the mobilised corporal limb (along the bisector) and the vertical/horizontal was measured [6].

### 2.4. Statistical Analysis

A post hoc sample size calculation was performed with the software package G*Power 3.1.9.7. (Heinrich Heine-Universität Düsseldorf, Düsseldorf, Germany) using a multivariate analysis of variance (MANOVA) test.

Sex differences in descriptive variables were compared using the *t*-test for independent samples. Differences between right and left limbs in ROM tests were compared using the paired-samples *t*-test. Additional effect sizes were estimated using Hedge’s g with values reported as trivial (<0.2), small (0.2 to 0.59), moderate (0.6 to 1.19), large (1.20 to 2.00), or very large (2.00 to 3.99).

A k-means cluster analysis was performed to determine a cut-point value and classify the children into those with low or high ROM, and normal (normal sagittal spinal alignment) or increased (sagittal spinal misalignment) sagittal thoracic and lumbar curve. Differences between the dependent variables of scalar type (thoracic and lumbar) and the independent variables of nominal type (ADF-KF, HE, and HF-KE) were performed using univariate (ANOVA) and multivariate (MANOVA) analysis of variance for males and females separately. In addition, effect size using partial eta squared (η^2^) was used to estimate the significance of differences and interpreted as small 0.01–0.05; medium 0.06–0.13; large > 0.14 according to Cohen. Finally, multivariate prediction (age, stature, weight mass, BMI, and ROM) was examined using binary logistic regression for males and females separately, with odds ratio (OR) values reported as a poor predictor of sagittal spinal misalignment (OR < 1); small predictor (OR from 1 to 1.25; medium predictor (OR from 1.25 to 2); and large predictor (OR ≥ 2).

## 3. Results

Test–retest reliability (20 children, 2 assessment sessions 24 h apart) in a preliminary double-blind study was higher than 0.92 for all variables (sagittal spine curves ICC: curve 0.93 to 0.98; ROM ICC, 0.95 to 0.98).

Table 1 shows that the female children had lower values of thoracic curve in SSP, and higher values of lumbar curve in RSP than male children (*p* = 0.000; g = moderate). In addition, female children had lower levels of kyphosis or lumbar inversion in SSP and MTFP than male children (*p* ≤ 0.001; g = moderate).

A k-means cluster analysis determined a cut-point (low vs. normal ROM, and normal vs. increased curve) for ADF-KF at 35° and 37°, EH 11° and 17°, and HF-KE at 65° and 77°; and for the thoracic spine in SSP > 42° and >31°, in RSP > 35° and >33°, and in MTFP > 53° and >53°, and for the lumbar spine in SSP > 17° and >3°, in RSP > 29° and >33°, and in MTFP > 29° and >25° in male and female children, respectively.

The comparison between the group with normal and the group with increased spinal curve in different postures (Table 2) showed that the group with increased thoracic curve in SSP (*p* = 0.026; g = small) and RSP (*p* = 0.048; g = small) had lower values of EH than the group with normal thoracic curve in male children. In the same sample, the group with increased thoracic curve in MTFP (*p* = 0.003; g = moderate) had lower HF-KE values than the group with normal thoracic curve in male children. 

In contrast, increased lumbar curve in the SSP (*p* = 0.031; g = large) and MTFP (*p* = 0.004; g = moderate) groups had higher HF-KE values than the group with normal lumbar curve in male children. In the female children, only in the group with increased lumbar curve in RSP (*p* = 0.005; g = moderate) were lower HE values found than in the group with normal lumbar curve.

The statistical power of the sample was retrospectively calculated for the variables where significant differences were found between the classification groups using the input parameters sample size (Table 2), alpha level *p* < 0.05, effect size (g = −1.215 to 0.720; Table 2) for multivariate analysis of variance (MANOVA) test. The analysed variables yielded a statistical power of 0.73 for HE in the classification of thoracic curve in SSP and 0.80 for HE in the classification of thoracic curve in RSP in the male children. A value of 0.88 was obtained in the classification of lumbar curve in RSP among the female children. The statistical power in HF-KE was 0.97 in the classification of thoracic curve in MTFP; and 0.99 in the classification of lumbar curve in SSP and 0.90 in the classification of lumbar curve in SSP MTFP in male children.

Multivariate analysis revealed differences between the means of HE (F = 3.052; *p* = 0.009; η^2^ = large) and HF-KE (F = 2.400; *p* = 0.034; η^2^ = large) and those of thoracic and lumbar curves. Univariate analysis revealed differences between the means of ADF-KF and lumbar curve in RSP (F = 5.207; *p* = 0.025; η^2^ = small); between the means of HE and thoracic curve in SSP (F = 8.923; *p* = 0.004; η^2^ = medium) and lumbar curve in RSP (F = 5.9021; *p* = 0.017; η^2^ = medium); and between the means of HF-KE and thoracic curve in SSP (F = 5.282; *p* = 0.024; η^2^ = small).

Finally, Table 3 shows that HE (*p* ≤ 0.028; OR ≥ 1.066) predicted thoracic curve classification in SSP (small OR) and RSP (medium OR) for male, and lumbar curve classification in RSP (female), while HF-KE (*p* ≤ 0.006; OR ≥ 1.089 (small)) predicted thoracic curve classification in MTFP and lumbar curve classification in SSP and MTFP (male).

## 4. Discussion

This is the first study to investigate ROM as risk factors associated with sagittal spinal misalignment in the habitual everyday posture in children. In general, the results of this study showed that HE had an influence on sagittal spinal alignment in SSP and RSP, and HF-KE had an influence on sagittal spinal alignment in SSP and MTFP. The ROM showed a greater influence on sagittal spinal alignment in male than in female children. 

Gender was found to have an influence on the results of this study as well as previous studies [16,21,22,23]. Consistent with our findings, male children showed more thoracic kyphosis in SSP [16,24] and RSP [22,23] and more lumbar inversion or lumbar kyphosis in SSP [24] and MTFP [16] than female children.

Several studies have shown the development of the sagittal spinal alignment in the standing posture. Poussa et al. [25] studied the development of spinal posture in a cohort of 1060 children aged 11 to 22 years. Their data indicated that thoracic kyphosis was more pronounced in males at all ages and that it increased with age in males but not in females. A longitudinal study observing the development of sagittal spinal alignment in children aged 5–16 years showed that thoracic kyphosis decreased with age in girls but not in boys [26]. On the other hand, poor or faulty posture in SSP, which is a very common posture in children, or RSP can also cause an increase in thoracic kyphosis [26] and lumbar kyphosis [27]. 

In contrast, female children showed a greater increase in lumbar lordosis or lumbar hyperlordosis in RSP than male children. Several authors also observed greater lumbar lordosis in female children of all ages [16,23,25]. In general, lordosis increases with age, with the highest values found in adults [28] and the trend decreasing after the seventh decade of life [29,30]. The higher lumbar curve in female children could be explained by the structural phylogenetic adaptations of the female spine [31]. All these differences between the sexes can be explained by the growth spurt at puberty. The completion of growth and mineralization of the secondary ossification centre of the vertebra [26,31] and structural changes of the spine [29,30] are more premature in female children. The peak increase in lumbar lordosis was observed in 11- to 15-year-old female subjects [31], which is consistent with the age of the female children in the present study. The female children in the present study had higher weight and BMI than the male children. Previous research studies investigating the lumbar curve in 405 children (210 girls and 195 boys) aged 10 to 13 years have shown that body weight in girls is associated with increased lumbar lordosis in RSP [22].

Limitation of ROM due to muscle tightness is one of the factors associated with sagittal spinal misalignment. Tightness of hamstrings [32,33,34] and iliopsoas [35] has been observed particularly frequently in children. Different authors have described different mechanisms to explain this decrease in muscle extensibility. The long and sedentary posture in the chair (at school and in everyday life while watching TV, using the computer, and playing on the tablet) may be a factor contributing to muscle tightness [36] by placing the hip, knee, and ankle in flexion at approximately 90°, shortening the soleus, iliopsoas, and hamstrings, and increasing muscle stiffness. Several authors have described a theoretical framework for the cause of increased passive stiffness [37,38,39]. The long and sedentary posture in the chair leads to a restriction of muscle metabolism with detrimental effects on the regulation of inflammation, oxygenation of muscle tissue, and blood flow [37,38]. In addition, decreased muscle metabolism appears to trigger a reactive imbalance in the muscle cell [38,40] that promotes the spontaneous formation of weak but long-lasting cross-bridges between myosin heads and actin filaments [41] and causes an increase in passive muscle stiffness [39]. Similarly, longitudinal development of the extremities during the pubertal growth spurt results in reduced muscle extensibility [32].

In the present study, muscle tightness was defined as being ≤35° and ≤37° for ADF-KF, ≤11° and ≤17° for HE, and ≤65° and ≤77° for HF-KE in male and female children, respectively. Gender differences have been demonstrated in previous studies [34,35], in which females had a higher ROM than male children. However, previous studies have not established reference or cut-point values for ROM that distinguish those children who are at high risk for sagittal spine misalignment. To the author’s knowledge, this study established the first reference values for five types of sagittal spinal misalignments in children. In general, it has been consistently demonstrated that restricted ROM increases the thoracic and lumbar curves in children [42]. Depending on the posture adopted (sitting, standing, or trunk flexion), these muscles can influence the sagittal disposition of the pelvis and spinal curves [42]. In this sense, the muscles that attach to the hip showed an influence on the sagittal spinal alignment in the three postures studied.

One of the actions of the iliopsoas is anterior pelvic tilt, which leads to an increase in lumbar lordosis in a standing posture [10,11], especially when the hip and knee are in neutral position. This lumbar compensation was observed in the female children in this study. Participants with tightness of the iliopsoas are associated with high thoracic kyphosis in RSP. This biomechanical relationship between the hip and the lumbar spine is consistent with previous studies showing a high frequency of iliopsoas tightness [35], and lumbar hyperlordosis [43] have been reported in populations with similar demographic characteristics. Furthermore, spinal misalignments are also determined by posture and weakness of the hip extensors and trunk flexors [11].

Increased thoracic kyphosis in RSP has also been reported to be caused by tightness of the iliopsoas in male children in this study. A logical theoretical argument would be that iliopsoas tightness increases lumbar lordosis, and this maladjustment of the body’s center of gravity is usually compensated by an increase in thoracic kyphosis [11]. Nevertheless, this argument only applies to female children in the present study. From a comprehensive perspective, sagittal spinal misalignment has a multifactorial origin, and it may be that other factors such as a relaxed kyphotic postural disposition typical of children [11,16], the pectoralis minor and shoulder adductors tightness, and the thoracic extensors and trapezius (middle and lower portions) weakness cause thoracic hyperkyphosis [11] in RSP and SSP. In SSP, the latter explanation for increased thoracic kyphosis may be more applicable, as a long and sedentary chair posture that maintains the iliopsoas in a shortened length through hip and knee flexion contributes to the stiffness and shortening of the iliopsoas during 4.5 h [36]. 

This study found that increased thoracic hyperkyphosis for MTFP in male children is caused by hamstring tightness. Our findings are consistent with previous reports in different populations [6,12,13]. Mechanical restriction of hamstring tightness alters the sequence of movements of the lumbar-pelvic rhythm in the MTFP [4,5,6].

As for the hamstring, its tightness has also been associated with the lumbar curve at SSP and MTFP in male children [6,12,13]. Children with increased lumbar lordosis (sagittal spinal imbalance) had higher hamstring extensibility values than those with a normal lumbar curve in this study. Higher hamstring extensibility allows these children to have an anterior sagittal disposition of the pelvis, which consequently improves the alignment of the lumbar spine [6,12,13]. In contrast, slumped posture of children in SSP and MTFP, and the influence of a hamstring tightness leads to greater retroversion of the pelvis and inversion of the lumbar curve [8], which causes mechanical stress and/or microtrauma to the anterior intervertebral discs and the passive elements of the posterior part of the spine [44].

The assessment of static (SSP and RSP) and dynamic (MTFP) sagittal spinal alignment in this study should be complemented by an analysis of spinal balance. Spinal balance is a dynamic phenomenon that describes a situation in which the forces present are equal or none of the forces exceeds the sum of the others [45,46]. In this state, the actions of the agonist and antagonistic muscles of the spine, pelvis, and hip are minimal and therefore cause less stress on the joint tissues during everyday postures [45,46]. Spine balance also requires consideration of more or less efficient compensatory phenomena that occur under dynamic conditions [45], such as walking, getting up from a chair, or picking up a school bag from the floor. Spinal balance thus makes it possible to analyse the parameters of the maturation process of children and the changes in daily posture. Therefore, it would be interesting to assess spinal balance using the sagittal vertical axis in children with spinal misalignments. This assessment is a good parameter to analyse the spine balance of the same person over time, but not to compare the balance of individuals among themselves [45].

In the checklist of the STROBE statement with points that should be considered in reports on observational studies [14], the following limitations of the present study are pointed out: Lack of control for possible sources of bias (determination of sagittal spinal misalignment by k-means cluster analysis, sample and participant selection bias), a posteriori calculation of sample size, lack of internal validity (maturity of participants, equivalence of groups by gender or practice of a sporting activity) and external validity (novelty of the ISQUIOS programme intervention, case and control groups were formed by cluster analysis, convenience sampling, no control for multiple treatments of the same ISQUIOS programme). Despite these potential limitations, the current findings could help physical education teachers redesign the ISQUIOS programme to include stretching exercises for the iliopsoas and hamstring for schoolchildren with sagittal spinal misalignments.

Future studies should replicate the present study by including in their procedure the measurement of the extensibility of other muscles, such as the quadriceps [11,47] or hip rotators [48], which have also been associated with sagittal spine misalignments and back pain in schoolchildren. It is also recommended that the association between sagittal spine misalignment and increased risk of injury and back pain in children be investigated. Radiological analysis to measure sacral slope, sagittal vertical axis, and sagittal spinal alignment, and to identify spinal lesions may complement this study.

## 5. Conclusions

The reference values for restricted HE and HF-KE determined in this study are significantly associated with sagittal spine misalignments in male children, but not for the ankle ROM. Physical education teachers should include stretching exercises in the ISQUIOS programme to increase the extensibility of the iliopsoas (HE) and hamstrings (HF-KE) and prevent sagittal spinal misalignments in habitual everyday postures.

## Figures and Tables

**Table 1:** Age, anthropometry, spinal curves angles, and hip–knee range of motion outcome data (mean ± SD) of the children included in the study (*n* = 201).

Variables		Total (*n* = 201)	Male (*n* = 103)		Female (*n* = 98)		*p*-Value	Hedges’ g
Age (Years)		10.9 ± 0.7	10.9 ± 0.8		10.9 ± 0.7		0.811	−0.000 (trivial)
Stature (Centimetre)		148.4 ± 7.3	148.1 ± 7.6		148.8 ± 7.1		0.480	−0.094 (small)
Weight Mass (kg)		45.7 ± 11.6	44.5 ±11.2		46.9 ± 12.0		−2.368	−0.206 (small)
BMI (kg/m^2^)		20.5 ± 4.3	20.0 ± 4.1		21.0 ± 4.5		0.083	−0.231 (small)
Thoracic Curve (Degrees)	SSP *	38.2 ± 11.9	41.9 ± 10.0		34.3 ± 12.5		0.000	0.670 (moderate)
	RSP	37.4 ± 9.4	39.1 ± 9.4		35.6 ± 9.1		0.008	0.376 (small)
	MTFP	54.4 ± 11.8	55.4 ± 11.4		53.3 ± 12.1		0.197	0.178 (trivial)
Lumbar curve (Degrees)	SSP *	10.0 ± 11.0	13.3 ± 10.7		6.6 ±10.2		0.000	0.638 (moderate)
	RSP *	−34.6 ± 10.1	−31.7 ± 9.6		−37.7 ± 9.7		0.000	−0.619 (moderate)
	MTFP *	25.0 ± 8.0	26.8 ± 7.5		23.1 ± 8.2		0.001	0.640 (moderate)
			**RL**	**LL**	**RL**	**LL**		
ROM (Degrees)	ADF-KF	36.9 ± 6.8	36.6± 7.4	35.8 ± 6.9	38.1 ± 7.2	37.2 ± 6.6	0.138	−0.209 (small)
	HE	14.1 ± 7.3	13.2 ± 7.5	14.0 ± 7.3	14.4 ± 7.4	14.8 ± 7.6	0.308	−0.143 (trivial)
	HF-KE *	71.4 ± 9.9	68.7 ± 9.1	67.3 ± 9.1	75.6 ± 9.8	74.4 ± 9.7	0.000	−0.749 (moderate)

BMI, body mass index; ROM, range of motion; RL, right limb; LL, left limb; SSP, slump sitting posture; RSP, relaxed standing posture; MTFP, maximum trunk forward flexion posture; ADF-KF, ankle dorsiflexion with knee flexed range of motion; HE, hip extension range of motion; HF-KE, hip flexion with the knee extended range of motion. Significant differences with moderate or larger effect size in sex are denoted by *.

**Table 2:** Range of motion outcome data (mean ± SD) for normal and increased spinal curve in different postures according to gender.

		Males (*n* = 103)					Females (*n* = 98)				
**Ankle Dorsiflexion with Knee Flexed Range of Motion (Degrees)**											
**Curve**		**Normal**	**N**	**Misalignment**	**N**	***p*-Value**	**Normal**	**N**	**Misalignment**	**N**	***p*-Value**
Thoracic	SSP	36.9 ± 6.4	56	35.3 ± 7.4	47	0.220	37.6 ± 6.8	36	37.6 ± 6.7	61	0.994
	RSP	36.2 ± 6.2	36	36.2 ± 7.3	67	0.973	37.7 ± 7.4	36	37.6 ± 6.3	61	0.920
	MTFP	36.2 ± 7.6	40	36.1 ± 6.5	63	0.948	36.6 ± 6.0	47	38.5 ± 7.2	50	0.165
Lumbar	SSP	36.0 ± 6.7	62	36.4 ± 7.4	41	0.795	37.6 ± 6.9	31	37.6 ± 6.6	66	0.963
	RSP	37.4 ± 6.6	37	35.5 ± 7.1	66	0.167	39.8 ± 6.4	23	36.9 ± 6.6	74	0.076
	MTFP	35.9 ± 7.6	64	36.7 ± 5.7	39	0.523	37.1 ± 6.3	65	38.8 ± 7.3	32	0.213
**Hip Extension Range of Motion (Degrees)**											
**Curve**		**Normal**	**N**	**Misalignment**	**N**	***p*-Value**	**Normal**	**N**	**Misalignment**	**N**	***p*-Value**
Thoracic	SSP	15.0 ± 7.1	56	11.8 ± 7.1	47	0.026 *	14.5 ± 8.3	37	14.6 ± 6.6	61	0.993
	RSP	15.3 ± 8.2	36	12.2 ± 6.5	67	0.048 *	14.9 ± 7.9	36	14.4 ± 6.9	62	0.728
	MTFP	13.8 ± 6.8	40	13.4 ± 7.5	63	0.767	14.4 ± 7.2	48	14.8 ± 7.4	50	0.776
Lumbar	SSP	14.3 ± 7.5	62	12.4 ± 6.6	41	0.204	14.7 ± 6.7	31	14.5 ± 7.6	67	0.897
	RSP	14.3 ± 7.5	37	13.1 ± 7.1	66	0.439	18.3 ± 7.6	23	13.5 ± 6.8	75	0.005 *
	MTFP	13.1 ± 7.4	64	14.4 ± 6.8	39	0.339	13.8 ± 6.6	65	16.2 ± 8.4	33	0.121
**Hip Flexion with Knee Extended Range of Motion (Degrees)**											
**Curve**		**Normal**	**N**	**Misalignment**	**N**	***p*-Value**	**Normal**	**N**	**Misalignment**	**N**	***p*-Value**
Thoracic	SSP	69.1 ± 8.5	56	66.6 ± 9.3	47	0.150	75.6 ± 10.2	37	74.5 ± 9.1	61	0.606
	RSP	68.6 ± 8.7	36	67.6 ± 9.1	67	0.573	77.0 ± 10.4	36	73.7 ± 8.8	62	0.096
	MTFP	71.4 ± 10.1	40	65.8 ± 7.4	63	0.003 *	75.8 ± 9.2	48	74.1 ± 9.8	50	0.400
Lumbar	SSP	66.4 ± 8.1	62	77.2 ± 10.1	31	0.031 *	70.3 ± 9.7	41	73.9 ± 9.1	67	0.098
	RSP	70.0 ± 8.7	37	66.8 ± 8.9	66	0.092	74.3 ± 7.3	23	75.1 ± 10.1	75	0.700
	MTFP	66.0 ± 8.7	64	71.2 ± 8.4	39	0.004 *	75.3 ± 9.7	65	74.3 ± 9.2	33	0.635

SSP, slump sitting posture; RSP, relaxed standing posture; MTFP, maximum trunk forward flexion posture. Normal: normal sagittal spinal alignment; Misalignment: sagittal spinal misalignment; Significant differences in range of motion are denoted by *.

**Table 3:** Hip range of motion as predictive of sagittal spine misalignments in male and female children.

Predictors	Sample	Curve	Posture	Odds Ratio (OR) ^1^	Standard Error	95% CI	*p*-Value
HE	Male	Thoracic	SSP	1.066 (Small)	0.174	0.886 to 0.993	0.028
	Male	Thoracic	RSP	1.861 (Medium)	0.207	0.885 to 0.998	0.003
	Female	Lumbar	RSP	1.694 (Medium)	0.210	0.954 to 1.088	0.012
HF-KE	Male	Thoracic	MTFP	1.095 (Small)	0.033	0.856 to 0.973	0.005
	Male	Lumbar	SSP	1.094 (Small)	0.032	1.028 to 1.165	0.005
	Male	Lumbar	MTFP	1.089 (Small)	0.025	1.021 to 1.126	0.006

SSP, slump sitting posture; RSP, relaxed standing posture; MTFP, maximum trunk forward flexion posture; HE, hip extension range of motion; HF-KE, hip flexion with knee extended range of motion; CI, Confidence Interval. ^1^ OR, Odds Ratio; OR < 1: poor predictor of LBP; OR from 1 to 1.25: small predictor; OR from 1.25 to 2: medium predictor; OR ≥ 2: large predictor.

## Data Availability

The data sets used and analysed during the current study are available from the first or corresponding author on reasonable request.
